# Syndromic Surveillance during Pandemic (H1N1) 2009 Outbreak, New York, New York, USA

**DOI:** 10.3201/eid1709.101357

**Published:** 2011-09

**Authors:** Marlena Gehret Plagianos, Winfred Y. Wu, Colleen McCullough, Marc Paladini, Joseph Lurio, Michael D. Buck, Neil Calman, Nicholas Soulakis

**Affiliations:** Author affiliations: New York City Department of Health and Mental Hygiene, New York, New York, USA (M.G. Plagianos, W.Y. Wu, C. McCullough, M. Paladini, M.D. Buck, N. Soulakis);; Institute for Family Health, New York (J. Lurio, N. Calman)

**Keywords:** viruses, influenza, outbreaks, surveillance, pandemic (H1N1) 2009, New York, emergency department, influenza-like illness, respiratory infections, United States, dispatch

## Abstract

We compared emergency department and ambulatory care syndromic surveillance systems during the pandemic (H1N1) 2009 outbreak in New York City. Emergency departments likely experienced increases in influenza-like-illness significantly earlier than ambulatory care facilities because more patients sought care at emergency departments, differences in case definitions existed, or a combination thereof.

Health departments perform syndromic surveillance to provide early warning of emerging outbreaks and provide situational awareness for ongoing outbreaks to help characterize magnitude and geographic scope of outbreaks over time. The New York City (NYC) Department of Health and Mental Hygiene, New York, New York, USA, conducts syndromic surveillance by using emergency department (ED) visits (*1**,**2*) and electronic health record data from ambulatory clinics in its Primary Care Information Project (*3*) and the Institute for Family Health (IFH), a network of community health centers (*4**,**5*).

The pandemic (H1N1) 2009 outbreak was first detected in NYC through traditional surveillance, a report of increasing influenza-like illness (ILI) at a high school in Queens on April 24 (*6*). After this report, Department of Health and Mental Hygiene syndromic surveillance detected citywide increases in patients with ILI seeking care at EDs. To assess the performance characteristics of ambulatory-based syndromic surveillance, we performed retrospective analyses to determine if the ambulatory-based system was also able to detect an increase in ILI during the spring 2009 outbreak, and, if so, determine whether it provided earlier notification or greater magnitude of detection compared with ED data.

## The Study

**Figure 1:**
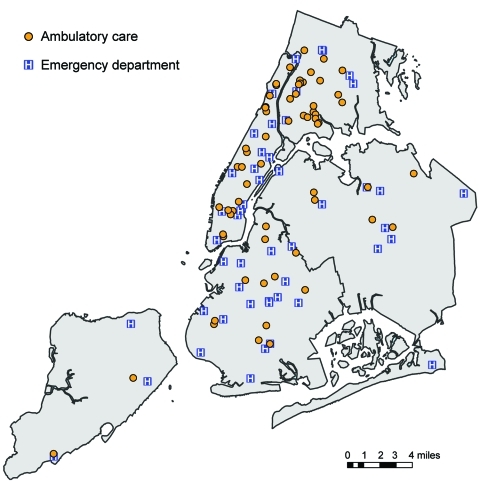
Locations of ambulatory care facilities and emergency departments used in analysis of syndromic surveillance of pandemic (H1N1) 2009, New York, New York, USA, May 2009.

Ambulatory surveillance data originated from 9 IFH facilities located in Manhattan and the Bronx and 49 primary care practices enrolled in the Primary Care Information Project and located throughout NYC. ED surveillance data originated from 50 EDs across the city. ED and ambulatory care facilities were similarly distributed (Figure 1). EDs had a high volume of patients (mean 247/day, mean age 34 years). Ambulatory care facilities saw fewer patients (mean 34.5/day, mean age 33 years).

ILI case definitions were based on previous correlations to seasonal influenza and differed slightly between systems. Within ambulatory clinics, ILI was defined as presence of fever (either measured temperature >99.9°F, or fever as a reason for visit) plus reason for visit of cough, “flu” or influenza, or ILI-related International Classification of Diseases, 9th Revision, encounter diagnosis (codes 079.99, 466.0, 487.1, 382.00, 465.9). The ED ILI case definition was based on a chief report of fever plus sore throat or cough, or chief complaint mentioning influenza.

For both systems, we calculated the percentage of ILI visits (number of ILI-related visits/total number of encounters) at each facility on weekdays (weekends were excluded because many ambulatory clinics were closed) and determined the first day each facility experienced an increase in the percentage of ILI visits, on the basis of 28-day moving averages and *z*-score. A significant increase was defined as a z-score >2 (*7*).

ED surveillance showed elevated ILI activity in 2 distinct phases during the spring 2009 pandemic (H1N1) 2009 outbreak (*8*). We counted the number of days during April 24–May 8 (representing the first phase of elevated ILI activity) when each facility experienced its first increase in ILI visits and the number of days during May 14–June 4 (representing the second phase) when each facility experienced its next increase in ILI visits. Because not all facilities experienced increases in ILI, we used the survival analysis method of the log-rank test to compare time to significant increase in ILI visits between EDs and ambulatory clinics. We repeated the analysis for each borough to assess potential differences within boroughs in the timing between systems to initial ILI increase. To compare the magnitudes of the signals, we used the Wilcoxon matched-pairs test to compare facility *z*-scores between systems.

**Figure 2:**
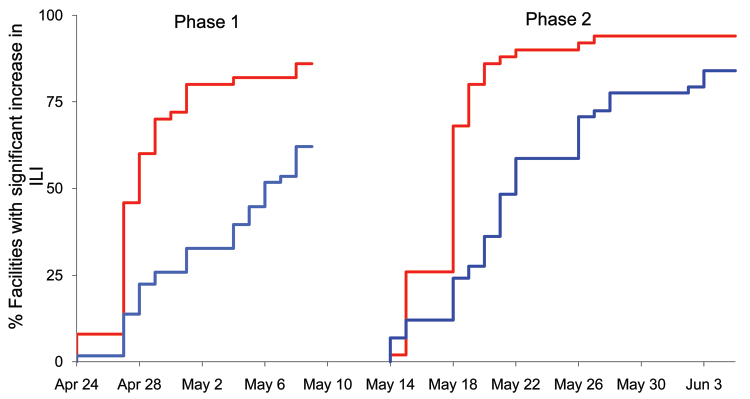
Percentage of emergency departments (red lines) and ambulatory clinics (blue lines) with substantial increases in patients with influenza-like illness (ILI) during phases 1 and 2 of pandemic (H1N1) 2009, New York, New York, USA, spring 2009.

Before April 24, syndromic surveillance data from both systems had shown decreasing levels of ILI (*8*). The survival curves (Figure 2) show that in the first phase of the pandemic (H1N1) 2009 outbreak, most EDs rapidly experienced noticeable increases in ILI, whereas the increase in ILI at ambulatory clinics occurred in fewer sites and was more gradual. During the second phase, most EDs immediately experienced substantial increases in ILI. Although more ambulatory facilities experienced a substantial increase in ILI compared with the first phase, the response was again more gradual. The survival curves differed significantly by the log rank test (p<0.001). When the analysis was repeated for each borough, EDs experienced an increase before ambulatory clinics across all boroughs except Staten Island during the first phase and in all boroughs during the second phase (Table).

**Table:** Borough-specific results for syndromic surveillance during pandemic (H1N1) 2009 outbreak, New York, New York, USA, 2009*

Borough and pandemic phase	Median days to increase in visits for ILI		p value†
	ED	AC	
Phase 1: Apr 24–May 8			
All	4	12	<0.001
Bronx	5	12	0.045
Brooklyn	3	14	0.025
Manhattan	4	13	0.008
Queens	3	7	0.007
Staten Island	14	10	0.902
Phase 2: May 14–Jun 4			
All	4	8	<0.001
Bronx	1	6	0.004
Brooklyn	4	12	0.039
Manhattan	4	7	0.016
Queens	4	8	0.091
Staten Island	5	8	0.012

*ILI, influenza-like illness; ED, emergency department; AC, ambulatory care. †1-sided log rank test.

The magnitude of the signals’ *z*-scores were significantly greater at EDs during the first phase (p = 0.004). However, they were not different during the second phase (p = 0.121).

## Conclusions

The results of this analysis confirm that ambulatory syndromic surveillance detected increases in ILI activity during both phases of the pandemic (H1N1) 2009 spring outbreak in NYC. However, the timeliness of detection appeared significantly earlier in EDs during both phases, and the magnitude of ILI signaling was significantly greater at the EDs during the first phase of the outbreak. During previous influenza seasons, the EDs and IFH ambulatory care facilities tracked well together (*4**,**5*).

There are several limitations worth noting. First, coverage of NYC EDs for syndromic surveillance is comprehensive (50 of 55 EDs), whereas the proportion of all NYC ambulatory clinics in this analysis is small. Better representation of NYC ambulatory clinics would possibly affect these results as there might be factors associated with electronic health record-based practices in the ambulatory surveillance system that resulted in the differences seen. Geographic distribution of the ambulatory clinics and EDs in this analysis is similar and differential sampling by location alone is unlikely to explain the differences.

Second, there are several EDs not participating in the syndromic surveillance network in eastern Queens where the pandemic (H1N1) 2009 outbreak first emerged. Given the proximity of these nonreporting hospitals to where the outbreak began, their inclusion might have altered the findings reported toward an earlier or stronger signal among EDs.

Third, ambulatory care facilities are able to triage telephone calls from patients and may have instructed patients with mild ILI symptoms to stay home. In addition, some ambulatory care patients may have had to wait several days between requesting a visit and receiving care. Either telephone triage or appointment delays could have reduced the number of ILI visits in these settings, which would not have been possible at EDs.

Fourth, the case definitions for ILI differ slightly between systems. The ED case definition is less specific because it includes patients reporting a chief complaint of “flu” alone, whereas the ambulatory care definition requires both febrile and respiratory symptoms and diagnoses. Thus, EDs may have detected greater increases in ILI because of higher sensitivity. Such an increase, especially in worried well patients, may have occurred during the outbreak, contributing to more ILI cases captured by the ED syndromic surveillance system.

Although earlier detection at EDs might be the result of persons choosing to go to EDs instead of ambulatory care clinics, it may have occurred because the less specific ED case definition was able to capture more events, or it may be a combination of these 2 factors. The findings reported here do not definitively demonstrate that ED syndromic surveillance is inherently timelier than ambulatory syndromic surveillance in detecting emerging influenza outbreaks. The heightened awareness of pandemic (H1N1) 2009 influenza during the spring 2009 outbreak may have affected the findings we reported. Further investigation during future outbreaks will help to better assess the innate abilities of the systems to provide early warning and situational awareness of emerging infectious disease outbreaks.
